# A Flexible Capacitive Pressure Sensor with Broad-Range High Sensitivity Based on 3D Porous Ionogel for Wearable Health Monitoring

**DOI:** 10.3390/mi17080983

**Published:** 2026-08-20

**Authors:** Yi Chen, Xuedan Xie, Yonghua Wang, Dan Liu

**Affiliations:** 1 State Key Laboratory of Extreme Environment Optoelectronic Dynamic Testing Technology and Instrument, North University of China, Taiyuan 030051, China; 2 Department of Electrical and Control Engineering, Shanxi Institute of Energy, Jinzhong 030600, China

**Keywords:** flexible pressure sensor, 3D porous ionogel, broad-range high sensitivity, wearable health monitoring

## Abstract

Flexible pressure sensors featuring high sensitivity, a broad detection range, and excellent stability are pivotal components for high-precision electronic skins and human health monitoring. To circumvent the limitations of existing sensors in maintaining high responsiveness across extensive pressure ranges, herein, a novel flexible capacitive pressure sensor is developed based on a 3D porous ionogel foam composite (IL/EG/PVA@MF) coupled with a planar electrode array. This device leverages the synergistic structural engineering of the 3D hyperelastic melamine foam (MF) skeleton and the pressure-regulated fringe-field distribution and iontronic interfacial polarization of the porous ionogel. Experimental evaluations demonstrate that the sensor achieves a high normalized sensitivity of 62.45 kPa^−1^ (2–10 kPa) and maintains reliable piecewise linear sensing performance across a broad working range of 0–50 kPa, accompanied by a rapid response time of within 8 ms. Furthermore, the sensor exhibits outstanding performance consistency after 6000 compression-release cycles at 50 kPa, verifying its good mechanical durability. In practical applications, the device can monitor diverse physiological signals with high fidelity, ranging from subtle radial artery pulses to large-scale joint movements and specific coughing patterns, underscoring its broad potential for integrated wearable systems and intelligent healthcare.

## 1. Introduction

With the rapid evolution of Industry 4.0, artificial intelligence, human–machine interaction, and remote healthcare systems, flexible pressure sensors have become important components in electronic skins, wearable health-monitoring platforms, and intelligent tactile interfaces [1,2,3,4,5]. In personalized healthcare, ideal pressure sensors are expected to detect both subtle physiological signals, such as radial artery pulses and respiratory airflow, and relatively large mechanical stimuli, such as joint bending and plantar pressure [6,7,8,9]. Among various transduction mechanisms, including piezoresistive, piezoelectric, triboelectric, and capacitive sensing, capacitive pressure sensors have attracted considerable attention because of their low static power consumption, good signal stability, high signal-to-noise ratio, and compatibility with flexible device integration [10,11,12,13].

To improve the sensitivity and working range of flexible capacitive pressure sensors, several structural and material-design strategies have been developed. Conventional solid polymer dielectric layers offer simple structures and stable capacitance output, but their high modulus and limited compressibility often result in low sensitivity and rapid response saturation under pressure [14,15]. Microstructured dielectric layers, including pyramids, microdomes, micropillars, and biomimetic patterns, can effectively enhance local deformation and contact-area variation, thereby improving low-pressure sensitivity [16,17]. However, these microstructures usually require template-assisted molding, photolithography, laser processing, or other sophisticated fabrication methods, which may increase fabrication complexity and limit scalable manufacturing. Porous dielectric structures based on sponges, foams, and aerogels provide large deformation space and high compressibility, making them promising for broad-range pressure sensing [18,19,20]. Nevertheless, conventional porous elastomers generally suffer from low dielectric constants and limited interfacial polarization, resulting in insufficient capacitance variation. Composite dielectric layers incorporating conductive nanomaterials, high-k fillers, ionic liquids, or ionogels have therefore been explored to enhance dielectric polarization and interfacial capacitance [21,22,23,24]. Despite these advances, it remains challenging to simultaneously achieve high normalized sensitivity, broad working range, fast response, long-term durability, and simple fabrication in one flexible capacitive sensor.

Ionogels are particularly attractive for capacitive/iontronic sensing because mobile ions can induce strong interfacial polarization at electrode/gel interfaces and improve pressure-induced capacitance variation [24]. Compared with water-rich conductive hydrogels, ionic-liquid-based ionogels generally show lower volatility, better dehydration resistance, and improved environmental stability [25,26,27]. In addition, three-dimensional porous frameworks can amplify pressure-induced deformation and modulate the effective dielectric environment above the electrodes. Therefore, integrating ionogels with compressible porous scaffolds provides a promising route for constructing high-performance flexible capacitive pressure sensors. However, many reported devices still rely on complex surface microstructuring or conventional planar electrodes, and the coupling between porous-framework deformation, fringe-field modulation, and iontronic interfacial polarization has not been fully exploited.

Herein, a flexible capacitive pressure sensor based on an IL/EG/PVA@MF porous ionogel foam and coplanar serrated-interdigital electrodes is reported. Different from previously reported capacitive sensors that mainly rely on either surface microstructuring or conventional porous elastomers, this device integrates three functional elements into one pressure-sensitive architecture. First, the commercial melamine foam (MF) provides a lightweight, highly porous, and recoverable scaffold for vertical air-gap compression and large deformation accommodation. Second, the IL/EG/PVA ionogel introduces mobile ions and enhances pressure-regulated interfacial polarization within the coplanar fringe-field region. Third, the serrated-interdigital electrode configuration enables a simple planar device structure while promoting local fringe-field modulation and electrode/ionogel contact sensitivity. This synergistic design avoids complex lithographic micro-patterning and helps balance sensitivity, working range, response speed, and mechanical durability. As a result, the proposed sensor exhibits a high normalized sensitivity of 62.45 kPa^−1^ in the medium-pressure region of 2–10 kPa, a broad working range of 0–50 kPa, fast response/recovery times within 8 ms, and stable capacitance output over 6000 compression-release cycles at 50 kPa. Furthermore, the sensor can monitor diverse physiological and motion signals, including finger bending, wrist movement, foot motion, breathing, coughing, and radial artery pulse waves, demonstrating its potential for wearable health monitoring and flexible human–machine interfaces.

## 2. Materials and Methods

### 2.1. Materials

Polyvinyl alcohol (PVA) was purchased from Aladdin. The ionic liquid 1-ethyl-3-methylimidazolium bis(trifluoromethylsulfonyl)imide ([EMIM][TFSI]) was obtained from Zhengzhou Alpha Chemical Co., Ltd. (Zhengzhou, China). Acetonitrile (AN, 99.0%) and ethylene glycol (EG, AR grade) were provided by Macklin and Sinopharm Chemical Reagent Co., Ltd. (Shanghai, China), respectively. Commercial MF, polyimide (PI) substrates, 3 M tape, conductive silver paste, and silver fibers were purchased from Alibaba Co., Ltd. (Hangzhou, China). All chemicals were used as received without further purification.

### 2.2. Fabrication of IL/EG/PVA-Loaded Porous Melamine Foam (IL/EG/PVA@MF)

The porous ionogel foam was fabricated using a vacuum impregnation and freeze-drying process. Pre-treatment of MF skeleton: Commercial MF was cut into 12 × 12 × 3 mm^3^ cubes and ultrasonically cleaned in a 1:1 (*w*/*w*) mixture of ethanol and deionized (DI) water for 30 min. The foams were then dried to ensure a rigid, moisture-free skeleton.

Preparation of IL/AN/EG/PVA precursor solution: 16 g of EG was mixed with 16 mL of DI water and stirred at 60 °C for 30 min. Subsequently, 2 g of PVA was added, and the temperature was raised to 95 °C for 1 h to ensure complete dissolution. After cooling to room temperature, 5 g of [EMIM][TFSI] and 5 g of AN were added dropwise and stirred for another hour to obtain a homogeneous IL/AN/EG/PVA precursor solution with moderate fluidity. In this precursor system, AN was used as a volatile co-solvent to reduce viscosity and facilitate uniform infiltration into the MF scaffold, whereas EG acted as a high-boiling plasticizing solvent to improve flexibility and help maintain ionic mobility in the PVA/[EMIM][TFSI] network.

Impregnation and freeze-drying: The pretreated MF membrane was fully immersed in the gel solution under vacuum for 30 min to remove trapped air and ensure complete pore infiltration. Excess solution on the surface was gently removed with lint-free paper. Finally, the samples were frozen at −85 °C for 12 h and freeze-dried at −50 °C for 24 h. During this process, water and most of the volatile AN were expected to be removed, while PVA, [EMIM][TFSI], and residual EG were retained within the MF scaffold, forming the final flexible IL/EG/PVA@MF porous ionogel foam dielectric layer.

### 2.3. Assembly of the Flexible Capacitive Pressure Sensor

**Figure 1:**
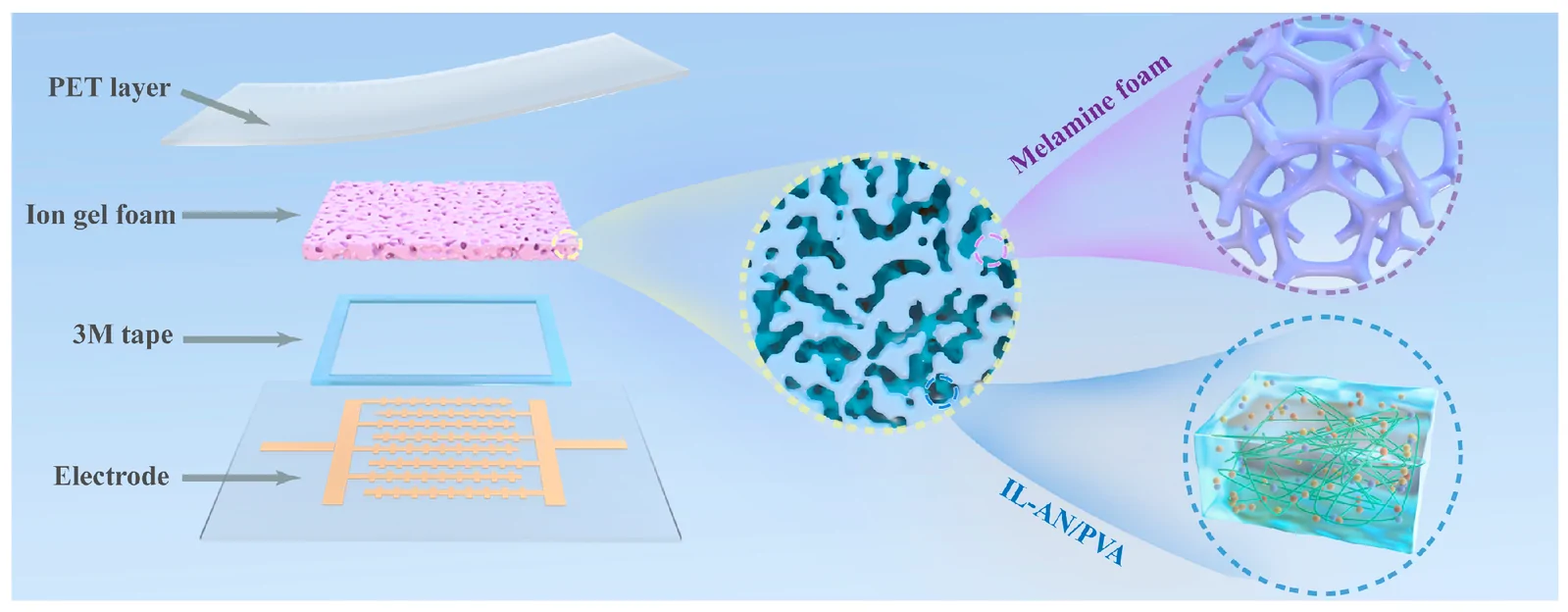
A schematic diagram of the flexible capacitive pressure sensor based on ionic gel foam.

The sensor assembly utilized a planar configuration with serrated-interdigital electrodes. Electrode Preparation: Symmetrical serrated-interdigital electrodes were fabricated on a PI substrate via screen printing using conductive silver paste. The electrodes were cured at 85 °C for 30 min. Device Integration: A square hollow 3 M tape (thickness: 0.2 mm; inner dimensions: 10 × 10 mm^2^) was applied over the electrode area to create a defined air gap. The IL/EG/PVA@MF porous ionogel foam dielectric layer was then placed on top of the spacer and secured at the corners. To avoid ambiguity, IL/AN/EG/PVA is used to describe the precursor solution before freeze-drying, while IL/EG/PVA@MF is used to denote the final porous ionogel foam dielectric layer after freeze-drying. The entire device was encapsulated with PI tape to form the final flexible sensing unit (Figure 1).

### 2.4. Characterization and Measurements

The cross-sectional morphology of the MF and IL/EG/PVA@MF porous ionogel foam was characterized using scanning electron microscopy (SEM). Chemical interactions were analyzed via Fourier-transform infrared spectroscopy (FT-IR). The mechanical stress–strain behavior was evaluated using a computer-controlled testing platform. Capacitance signals were recorded using a high-precision LCR meter (IM 3536, HIOKI E.E. CORPORATION, Ueda, Nagano, Japan) at a frequency of 1 kHz with an AC amplitude of 1 V. For human physiological monitoring, sensors were attached to the skin using medical-grade adhesive tape with informed consent from volunteers.

## 3. Results and Discussion

### 3.1. Structural Characterization and Qualitative Stress-Distribution Analysis

**Figure 2:**
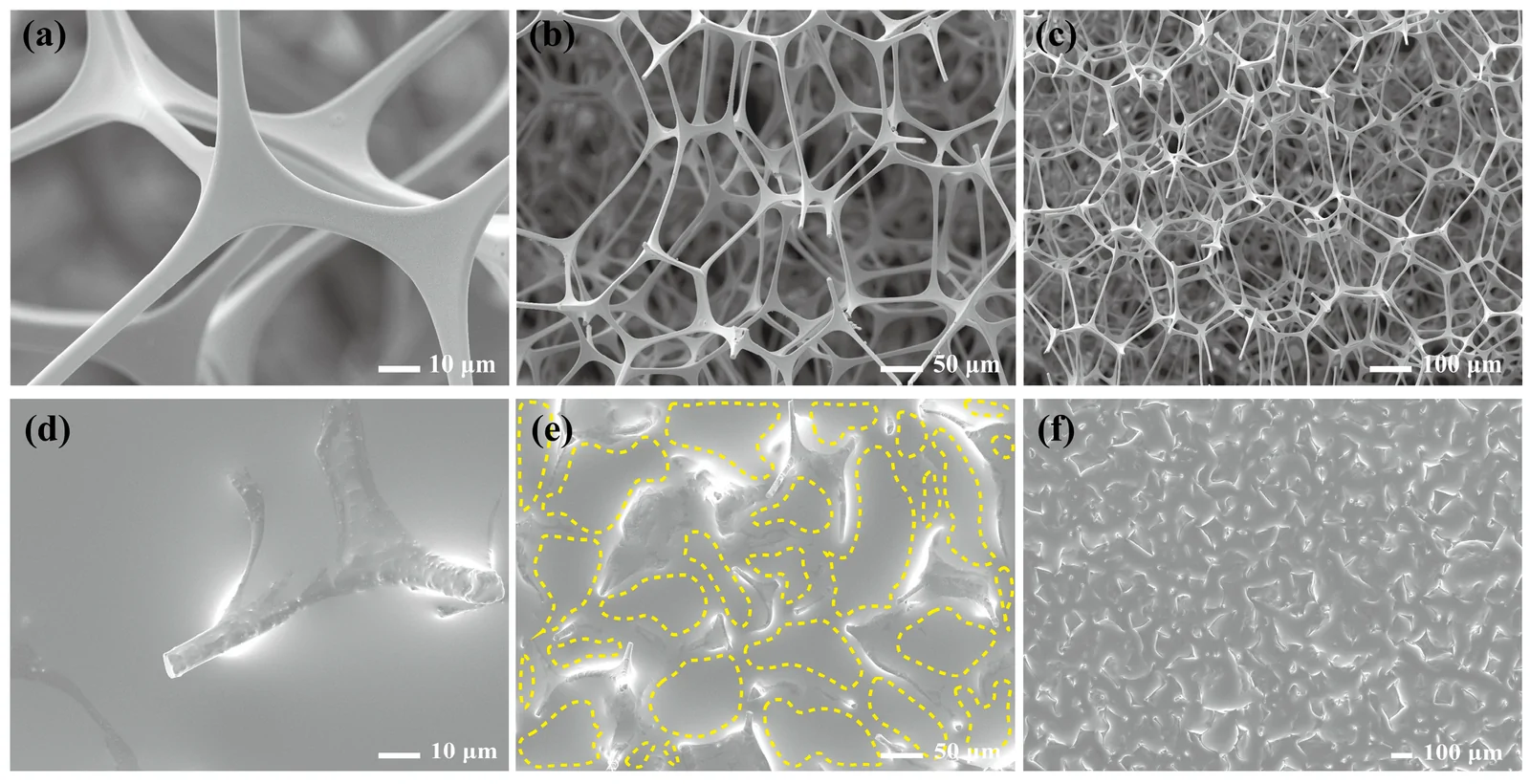
SEM images of MF at different magnifications: (**a**) 10 μm (**b**) 50 μm (**c**) 100 μm SEM images of MF loaded with ionic gel (IL/EG/PVA@MF) at different magnifications: (**d**) 10 μm (**e**) 50 μm (**f**) 100 μm.

The core of the high-performance pressure sensor lies in the synergistic effect between the hierarchical porous skeleton and the ionically conductive IL/EG/PVA porous ionogel. The SEM images of the pristine MF are shown in Figure 2a–c, which exhibit a 3D interconnected network with a high porosity of over 95%, featuring pore diameters ranging from 80 to 120 μm. This cellular structure provides low modulus and high compressibility, ensuring rapid reversible deformation under external pressure. After the vacuum infiltration and freeze-drying process, the IL/EG/PVA ionogel is uniformly loaded onto the MF struts, as depicted in Figure 2d–f. The SEM images show that the IL/EG/PVA ionogel wraps around the MF struts without fully clogging the macropores. The yellow box in Figure 2e highlights the gel matrix that fills the foam structure. Notably, the partial exposure of the MF framework endows the composite surface with a distinct micro-textured morphology (or micro-bumps) rather than a perfectly flat surface. While the gel matrix forms a continuous conductive network along the skeleton, the micro-textured surface of the IL/EG/PVA@MF porous ionogel foam suggests a limited initial contact with the coplanar electrodes, which may contribute to the low initial capacitance C_0_ together with the designed vertical air gap. Furthermore, some micro-sized air gaps (overexposed area in Figure 2d–f) remain in regions where capillary forces were insufficient. These residual air gaps and the inherent porosity of the MF are expected to participate in pressure-induced dielectric reconstruction, including air-gap compression, increased foam-electrode contact area, and replacement of low-permittivity air by the ionogel phase within the fringe-field region [28,29].

**Figure 3:**
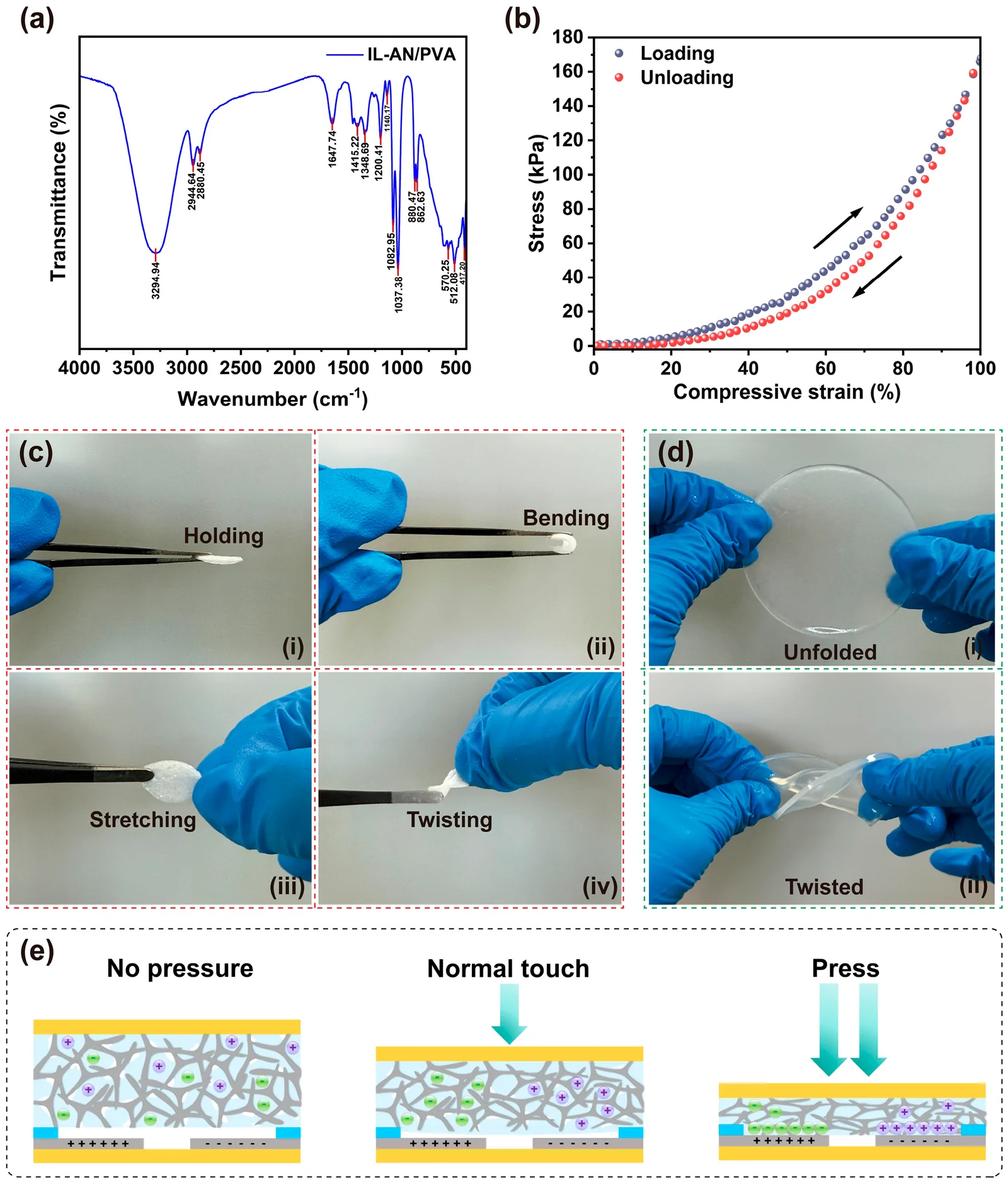
(**a**) FT-IR spectra of IL/EG/PVA gel. (**b**) Stress−strain behavior of the IL/EG/PVA@MF porous ionogel foam (upward for loading and downwardfor unloading). (**c**) Digital photographs of the IL/EG/PVA@MF porous ionogel foam under different mechanical deformations: (**i**) holding; (**ii**) bending; (**iii**) stretching; (**iv**) twisting. (**d**) Photographs of mechanical deformation of IL/EG/PVA gel: (**i**) unfolded; (**ii**) twisted. (**e**) A schematic illustration of the sensing mechanism. In the initial state, a vertical air gap exists between the ionogel foam and the interdigitated electrodes. Upon compression, the foam is deformed, reducing the gap and increasing the contact area between the electrodes and the ionogel, thereby altering the dielectric environment of the fringe electric field. Meanwhile, ion migration occurs at the interface and forms electric double layers, resulting in a change in capacitance (purple “+” spheres: cations; green “–” spheres: anions).

Figure 3a confirms the chemical interaction between the components through FT-IR spectroscopy analysis of the IL/EG/PVA ionogel. A broad characteristic peak at 3294.94 cm^−1^ is attributed to the stretching vibration of the hydroxyl group (–OH) in PVA [30], further broadened by hydrogen bonding with the [EMIM]^+^ cations [31]. The strong interfacial bonding between the PVA chains and the MF (via –OH and –NH_2_ interactions) suppresses ion leakage and enhances the mechanical stability of the composite dielectric layer [32].

The stress–strain analysis illustrated in Figure 3b further confirms the mechanical robustness and recoverability of the IL/EG/PVA@MF porous ionogel foam. The foam can withstand compressive strain up to 100% and recover its original shape after unloading, indicating good structural resilience. To quantitatively evaluate the mechanical hysteresis, the areas enclosed by the loading and unloading stress–strain curves were calculated. The integrated areas of the loading and unloading curves were 4789.73 and 4061.38 kPa%, respectively, corresponding to a hysteresis area of 728.35 kPa% and an energy-loss coefficient of 15.21%. This moderate hysteresis indicates acceptable elastic recovery of the porous ionogel foam under compression. Such mechanical resilience helps the sensor maintain a stable capacitance response during repeated pressure loading and unloading.

To ensure reliability in real-world scenarios, flexible pressure sensors must maintain functional stability under diverse mechanical loading modes, including bending, stretching, and compression. Consequently, the mechanical resilience of the dielectric layer is critical for long-term device performance. Figure 3c depicts the deformation behavior of the IL/EG/PVA@MF porous ionogel foam dielectric layer across a range of applied stresses. The observed structural integrity highlights the material’s exceptional flexibility and deformability, confirming its robust mechanical adaptability for wearable applications. Figure 3d illustrates the mechanical deformation of the IL/EG/PVA ionogel matrix before loading into the MF scaffold under various mechanical deformation conditions, clearly demonstrating its excellent elastic recovery and flexibility.

In addition, a qualitative stress-distribution analysis was provided in Figure S1 to visualize the stress-distribution tendency of the sensor structure under applied loading. The simulated stress field is mainly distributed within the active sensing region, suggesting that the patterned electrode/sensing area participates effectively in pressure transfer. This simulation is used only as qualitative support for the structural response, and no quantitative displacement-pressure relationship is inferred from it. The actual deformation behavior of the porous ionogel foam is instead discussed based on the experimentally measured stress–strain curve in Figure 3b.

### 3.2. Sensing Mechanism and Performance Analysis

Sensitivity is a paramount indicator for evaluating the performance of pressure sensors, reflecting their ability to respond to subtle mechanical stimuli. The sensitivity S is defined as follows:

(1) $$S=\frac{\delta (∆C\;/{\;C}_{0})}{\delta P}$$

where *C*_0_ is the initial capacitance without applied pressure, Δ*C* is the change in capacitance, and P is the applied pressure [33].

Figure 3e illustrates the sensing mechanism of the proposed flexible capacitive pressure sensor, whose response originates from the synergistic modulation of the coplanar fringe electric field, porous dielectric deformation, and iontronic interfacial capacitance. It should be noted that the device adopts coplanar serrated-interdigital electrodes rather than a conventional parallel-plate structure; therefore, the in-plane spacing between adjacent electrode fingers is fixed by the screen-printing pattern and does not decrease under external pressure. In the initial undeformed state, the IL/EG/PVA@MF dielectric layer maintains a loose porous architecture above the coplanar electrodes, and the designed vertical air gap leads to limited foam–electrode contact. As a result, the fringe electric field mainly passes through air and a small portion of the ionogel region, giving rise to a relatively low initial capacitance.

Upon pressure loading, the vertical air gap between the porous ionogel foam and the electrode surface is gradually compressed, and the IL/EG/PVA@MF layer conformally contacts more regions of the serrated-interdigital electrodes. Meanwhile, the elastic collapse of the MF skeleton reduces internal air voids, allowing low-permittivity air to be progressively replaced by the ionogel phase with higher dielectric/ionic polarizability. These changes increase the effective permittivity within the fringe-field region and enlarge the effective electrode/ionogel contact area, thereby enhancing the fringe-field capacitance. In addition, mobile ions in the IL/EG/PVA ionogel migrate and accumulate at the electrode/ionogel contact interfaces under the applied electric field, forming electric double layers (EDLs). The pressure-induced increase in contact area and shortening of ion-transport pathways further promote interfacial ionic polarization, contributing an additional EDL-related capacitance.

As the pressure further increases, most of the vertical air gap and internal voids are compressed, and the porous dielectric layer approaches a denser contact state. In this regime, the increase in effective contact area and dielectric reconstruction becomes slower, leading to a reduced slope of the capacitance response. Therefore, the proposed device can be regarded as a coplanar fringe-field capacitive sensor with pressure-enhanced iontronic interfacial capacitance. The high sensitivity and broad sensing range arise from the coupled effects of vertical air-gap compression, porous-framework deformation, effective-permittivity enhancement, enlarged electrode/ionogel contact area, and EDL-based interfacial capacitance modulation.

Accordingly, the total capacitance can be phenomenologically described by an equivalent parallel contribution from the fringe-field capacitance and the EDL-related interfacial capacitance:

(2) $$C_{total}\left(P\right)=C_{fringe}\left(P\right)+C_{EDL}(P)$$

where *C*_fringe_(*P*) represents the pressure-dependent fringe-field capacitance determined by the coplanar electrode geometry and the effective permittivity above the electrodes, while *C*_EDL_(*P*) denotes the equivalent EDL capacitance generated at the electrode/ionogel contact interfaces.

Since the electrode geometry is fixed, the pressure response is mainly governed by the increase in effective permittivity and contact area. Because the EDL contribution depends on ion migration and interfacial polarization, it is expected to be more pronounced at relatively low testing frequencies, whereas at higher frequencies the ionic polarization becomes less complete and the measured capacitance is increasingly dominated by the dielectric/fringe-field component.

**Figure 4:**
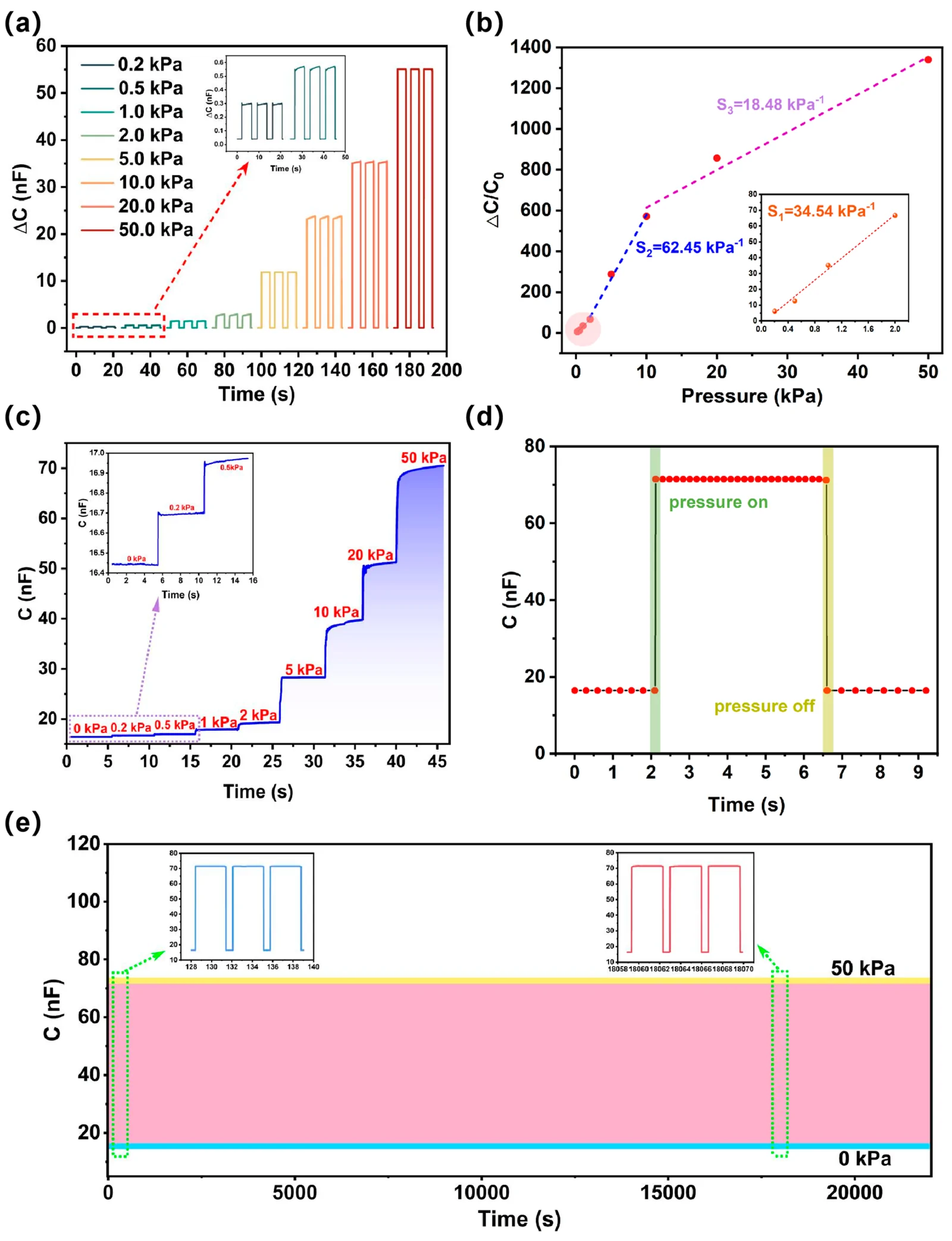
Performance testing of IL/EG/PVA@MF−based capacitive pressure sensor: (**a**) Pressure response range (inset is the pressure responses at 0.2 kPa and 0.5 kPa). (**b**) Sensitivity (inset is the sensitivity of S1). (**c**) Variation in capacitive output under continuous pressure gradients (inset is the capacitive output over 0–0.5 kPa). (**d**) Dynamic response time. (**e**) 6000-cycle stability test (insets are the initial and final pressure responses, respectively).

Figure 4a reveals that the IL/EG/PVA@MF-based sensor demonstrates a stable and continuous capacitance output over a broad pressure range of 0–50 kPa. As shown in Figure 4b, the sensitivity curve exhibits distinct tri-phasic linear characteristics. The pressure-response curve was obtained from three repeated measurements on one representative device (*n* = 3), and the sensitivities in the three pressure regions were determined by linear fitting of the averaged Δ*C*/*C*_0_–pressure curve. In the low-pressure region (0–2 kPa), the sensor shows an average sensitivity (S1) of 34.54 kPa^−1^, with an R^2^ of 0.9931 and an adjusted R^2^ of 0.9896. At this stage, the designed vertical air gap between the coplanar serrated-interdigital electrodes and the porous ionogel foam dominates the initial response. The ionogel/electrode interaction area is still limited, and only partial interfacial polarization is generated, resulting in a moderate capacitance increase. In the medium-pressure region (2–10 kPa), the sensitivity increases to a maximum value (S2) of 62.45 kPa^−1^, with an R^2^ of 0.9950 and an adjusted R^2^ of 0.9899. This enhancement is attributed to the rapid compression of the MF skeleton, reduction in the vertical air gap, expansion of the effective ionogel/electrode interaction area, modulation of the dielectric environment in the fringe-field region, and strengthened electric-double-layer interfacial capacitance. In the high-pressure region (10–50 kPa), the average sensitivity (S3) decreases to 18.48 kPa^−1^, with an R^2^ of 0.9823 and an adjusted R^2^ of 0.9646. This decrease can be attributed to the progressive densification and strain-stiffening of the porous foam. As the available deformation space becomes limited, the further increase in contact area and interfacial capacitance slows down, leading to a reduced but stable sensitivity over a broad pressure range. In addition, to make the capacitance response more transparent, the measured total capacitance and absolute capacitance change under different pressures are provided in Table S1. The initial total capacitance recorded by the LCR meter was 16.4411 nF for all pressure-response measurements. In Figure 4b, the normalized response Δ*C*/*C*_0_ was calculated using the effective initial capacitance after subtracting a constant baseline offset of 16.4 nF during data processing, i.e., *C*_0_ = 0.0411 nF. Therefore, the normalized response is mainly used to describe the relative pressure-induced capacitance variation in the device, while the absolute capacitance values in Table S1 provide a more direct basis for evaluating the sensor output. Although the MF skeleton approaches its densification limit and the compressibility decreases, the sensor maintains a reliable response, showing competitive performance among reported flexible capacitive sensors.

To further investigate the dynamic sensing capabilities in variable pressure environments, the device was subjected to a stepped pressure profile from 0 to 50 kPa. Each loading stage was held for 5 s to allow for steady-state output, while the transition interval between adjacent levels was minimized to within 100 ms. Figure 4c demonstrates that the capacitance response follows a well-defined stepwise increase, exhibiting excellent signal stability and the ability to distinguish discrete pressure increments.

Figure 4d shows the dynamic response of the sensor under an applied load of 20 kPa. Based on the original dynamic-response data, the capacitance transition from the baseline state to the loaded state occurred between two adjacent sampling points, from 2.104 s to 2.112 s, while the recovery transition occurred from 6.592 s to 6.600 s. Therefore, the response and recovery times were both estimated to be within 8 ms under the present data acquisition condition. Similar abrupt transitions were observed in the repeated measurements, and all calculated response and recovery times were no longer than 8 ms. It should be noted that this value is limited by the sampling interval of the measurement system, and the intrinsic response/recovery behavior of the sensor may be faster than the recorded time resolution. The response curve shows a clear rectangular profile without obvious overshoot or oscillation, indicating stable signal output under dynamic loading. This rapid response is mainly attributed to the compressible interconnected MF framework and the elastic IL/EG/PVA ionogel network, which facilitate fast pressure-induced deformation and recovery of the sensing layer.

Furthermore, Figure 4e and Table S2 show that the sensor demonstrates excellent long-term stability. During a 6000-cycle fatigue test at a high pressure of 50 kPa, the initial response amplitude was 55.0508 nF, and the response amplitude after 6000 cycles remained 55.0508 nF, corresponding to an amplitude retention of 100.0%, which demonstrated the capacitance signal remains highly consistent in both amplitude and waveform. This durability confirms the structural integrity of the IL/EG/PVA@MF composite and its potential for continuous monitoring in demanding wearable applications. The enhanced sensing mechanism of the IL/EG/PVA@MF sensor is a synergistic result of three coupled factors: the elastic collapse of the MF skeleton, the ionic transport optimization within the IL/EG/PVA matrix, and the dynamic modulation of air gaps. When pressure is applied, the compression of the vertical air gap, the densification of foam struts, and the increase in foam-electrode contact area significantly enhance the effective permittivity in the fringe-field region and promote EDL formation at the electrode/ionogel interfaces. This multi-factor coupling ensures high sensitivity and a rapid response speed across a wide operating range.

To further clarify the performance advancement, the key parameters of the proposed sensor were compared with representative flexible capacitive/iontronic pressure sensors based on microstructured dielectrics, porous scaffolds, and ionogel systems, as summarized in Table 1. The comparison includes sensitivity, working range, response/recovery time, cycling durability, and demonstrated applications. The proposed IL/EG/PVA@MF sensor shows a balanced sensing performance with high normalized sensitivity, a broad pressure range, fast response/recovery, and stable cycling durability, indicating the effectiveness of combining a porous ionogel foam with coplanar serrated-interdigital electrodes.

### 3.3. Practical Applications

Leveraging its high sensitivity, rapid response, and wide detection range, the developed IL/EG/PVA@MF-based sensor demonstrates significant potential in electronic skins and wearable health monitoring. To verify its practical utility, we conducted a series of experiments ranging from subtle touch recognition to complex physiological signal tracking.

#### 3.3.1. Motion and Touch Perception

**Figure 5:**
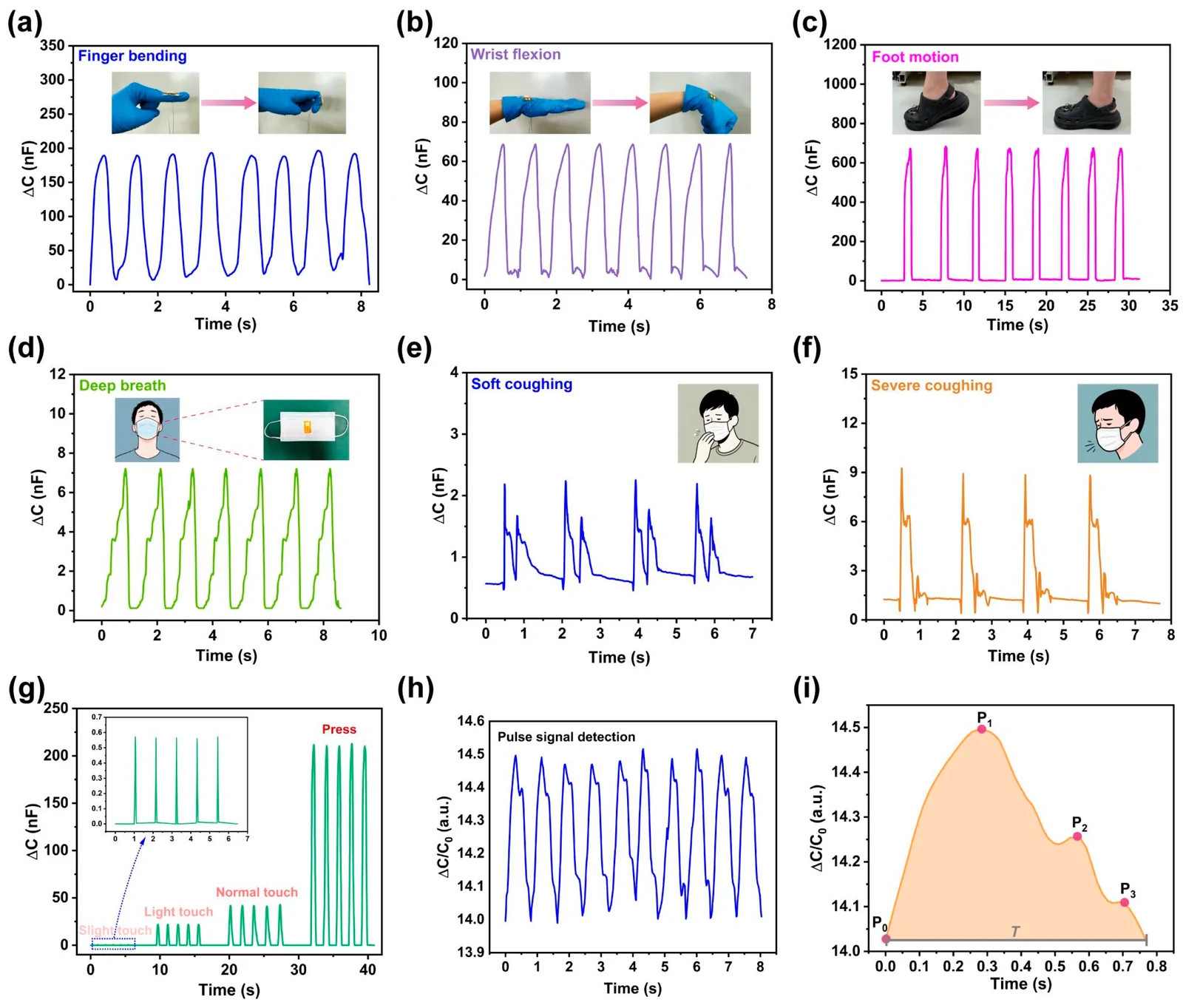
Monitoring results of sensors for different motion signals: (**a**) 90° single-finger flexion. (**b**) Press down on the wrist. (**c**) Foot exercises. Application testing of sensors in health management: (**d**) Deep breath. (**e**) Soft coughing. (**f**) Severe coughing. (**g**) Capacitance signal response for touch perception under conditions of slight contact, light touch, normal touch, and press (the illustration is a pressure response amplification diagram with a slight touch). Application test results of the sensor in human pulse monitoring: (**h**) Changes in capacitive signals during wrist pulse monitoring. (**i**) Single-pulse capacitive signal.

The sensor can accurately distinguish between various intensities of mechanical stimuli. Figure 5g compares the performance of the capacitance response, which correlates positively with the applied force, showing distinct signal amplitudes for slight contact (0.6 nF), light touch (21 nF), normal touch (43 nF), and firm pressing (210 nF). The high repeatability and low deviation of these signals confirm the sensor’s precision in fine-grained tactile sensing.

As visualized in Figure 5a–c, for joint motion monitoring, the sensor was attached to the proximal interphalangeal joint of the index finger and the radial extensor tendon of the wrist. When the finger performed a 90° bending-extension cycle, the output capacitance exhibited stable stepwise changes with a consistent amplitude of approximately 190 nF. Similarly, the sensor captured repetitive wrist flexion with high fidelity and signal overlap. Furthermore, in foot motion tests, the sensor recorded stable periodic signals during stepping, with an average capacitance of 675 nF and minimal latency. These results demonstrate the sensor’s mechanical adaptability and its ability to quantify the speed and magnitude of body movements.

#### 3.3.2. Respiratory and Coughing Pattern Recognition

Capturing respiratory patterns and coughing characteristics is vital for the early screening of respiratory diseases [36]. By integrating the sensor into a standard medical mask, we established a non-invasive monitoring system. Figure 5d–f display the dynamic response of the proposed sensor under typical respiratory activities, including rhythmic breathing and episodic coughing. During deep breathing, the sensor produced regular capacitance variations of approximately 7 nF. Notably, the sensor can distinguish between different coughing intensities. Soft coughing resulted in signals ranging from 0 to 2.5 nF with a smooth rise, whereas severe coughing triggered higher amplitudes (up to 9.3 nF) accompanied by high-frequency oscillations. These oscillations reflect the transient pressure fluctuations caused by high-velocity airflow and violent thoracic contractions. The ability to quantify these physiological differences provides a promising technical solution for at-home health management and symptom screening [43].

#### 3.3.3. High-Fidelity Pulse Waveform Analysis

Radial artery pulse monitoring is a cornerstone of cardiovascular health assessment [44]. The sensor was attached to the radial artery of a healthy female volunteer aged 33 years, and the heart rate during measurement was approximately 72 bpm. As shown in Figure 5h,i, the recorded waveform clearly exhibits three characteristic peaks: the primary wave (P_1_) associated with left ventricular ejection, the tidal wave (P_2_) related to arterial wave reflection, and the dicrotic wave (P_3_) associated with aortic valve closure. The radial augmentation index was calculated as AIr = P_2_/P_1_. Based on 10 consecutive pulse cycles from the same subject, the AIr value was calculated to be 50 ± 2%, where the value represents the mean ± standard deviation. It should be noted that radial AIr is affected by age, sex, heart rate, blood pressure, and waveform-analysis methods. Therefore, this single-subject result is not intended to provide a population-level cardiovascular assessment, but rather demonstrates that the proposed sensor can capture distinguishable radial pulse waveform features for potential wearable pulse-wave monitoring. This result indicates stable extraction of waveform-derived pulse parameters from repeated cycles under the tested condition.

It should be noted that the proposed device is positioned as a general-purpose multi-functional flexible pressure sensor rather than a pulse-diagnosis-specific biomedical device. The demonstrations of joint motion, respiration, coughing, and radial artery pulse monitoring are intended to verify its capability to detect diverse human-related mechanical signals over different pressure amplitudes. For arterial pulse monitoring, the measured waveform may be influenced not only by physiological arterial dynamics but also by the sensor size, attachment position, contact pressure, soft packaging, and the response characteristics of the capacitive sensing system. Therefore, the pulse signal in this work is mainly used as a proof-of-concept demonstration of wearable physiological pressure monitoring. Further validation involving multiple subjects, synchronized reference sensors, and systematic repeatability analysis will be required before the device can be used for specific biomedical diagnosis.

Benefiting from the synergistic integration of the compressible porous ionogel foam and coplanar serrated-interdigital electrodes, the sensor exhibits high normalized sensitivity, a broad working range, fast response/recovery behavior, and stable cycling performance, which make it suitable for detecting diverse human-related mechanical signals in wearable monitoring scenarios.

## 4. Conclusions

In summary, we have developed a high-performance flexible capacitive pressure sensor based on an IL/EG/PVA@MF porous ionogel foam and serrated-interdigital electrodes. The synergistic effect of the hierarchical porous MF, the pressure-regulated fringe-field distribution, optimized ionic interfacial polarization, and dynamic modulation of the vertical air gap and internal air voids enables a maximum sensitivity of 62.45 kPa^−1^ (2–10 kPa) and a wide range of 0–50 kPa. The sensor exhibits a rapid response time of within 8 ms and maintains structural integrity over 6000 high-pressure cycles. Successful demonstrations in monitoring human motion, respiratory patterns, and arterial pulses highlight its versatility for smart electronic skins and integrated storage-sensing wearable systems.

## Figures and Tables

**Table 1:** Comparison of key performance with reported flexible capacitive/iontronic pressure sensors.

Sensing Material/Structure	Sensor Type	Sensitivity	Working Range	Response/Recovery Time	Durability	Reference
Graded intrafillable ionic microstructure	Iontronic capacitive	Smin > 220 kPa^−1^	0.08 Pa–360 kPa	<10 ms response	5000 cycles at 300 kPa	Bai et al. [34]
Ionic-liquid-loaded high-porosity PU foam	3D foam iontronic capacitive	9280 kPa^−1^, 2–20 kPa; 1811.4 kPa^−1^, 20–60 kPa; 627.7 kPa^−1^, 60–114 kPa	up to 114 kPa	10/28 ms	5000 compression-release cycles	Liu et al. [35]
Two-scale random microstructured ionic gel film	Iontronic capacitive	12.8 kPa^−1^	0–1 MPa	20/30 ms	>10,000 compression-release cycles	Wu et al. [36]
Laser-induced gradient micropyramids + ionic layer	Iontronic capacitive	33.7 kPa^−1^	0–1700 kPa	6/11 ms	4500 cycles at 800 kPa	Yang et al. [37]
h-BN-assisted printable ionic film	Screen-printed iontronic capacitive	Smin > 261.4 kPa^−1^	0.05–450 kPa	15/23 ms	>5000 cycles at 400 kPa	Yang et al. [38]
Porous ionic gel foam with planar electrodes	Planar supercapacitive	1126.96 kPa^−1^	up to 300 kPa	25 ms response	6000 cycles	Tang et al. [39]
3D network spacer + ionic gel + MXene electrode	Iontronic capacitive	87.4 kPa^−1^, 400–1000 kPa	400–1000 kPa	46 ms response	10,000 cycles at 600 kPa	Xu et al. [40]
Microbubble-fabricated porous ionogel	Porous ionogel capacitive	684.4 kPa^−1^	~1 MPa	92/84 ms	4000 cycles at 200 kPa	Yang et al. [41]
NH_4_HCO_3_ in situ pore-forming porous ionic gel	Porous iontronic capacitive	25.3 kPa^−1^	0–1000 kPa	60/80 ms	1000 cycles, <3% degradation	Li et al. [42]
IL/EG/PVA@MF porous ionogel foam + serrated-interdigital electrode	Flexible capacitive	34.54 kPa^−1^, 0–2 kPa; 62.45 kPa^−1^, 2–10 kPa; 18.48 kPa^−1^, 10–50 kPa	0–50 kPa	within 8 ms	6000 cycles at 50 kPa	This work

## Data Availability

The original contributions presented in this study are included in the article/Supplementary Material. Further inquiries can be directed to the corresponding author.

## Supplementary Materials

The following supporting information can be downloaded at https://www.mdpi.com/article/10.3390/mi17080983/s1, Figure S1: Qualitative stress-distribution analysis of capacitive pressure sensors under different pressure simulations: (a) *p* = 0.5 kPa, (b) *p* = 2.0 kPa, (c) *p* = 10.0 kPa, (d) *p* = 50.0 kPa. Table S1. Absolute capacitance output and normalized capacitance response under different pressures. Table S2. Capacitance parameters before and after 6000 compression-release cycles.

## Abbreviations

The following abbreviations are used in this manuscript:

SNR	Signal-to-noise ratio
MF	Melamine foam
PVA	Polyvinyl alcohol
[EMIM][TFSI]	1-Ethyl-3-methylimidazolium bis(trifluoromethylsulfonyl)imide
AN	Acetonitrile
EG	Ethylene glycol
PI	Polyimide
DI	Deionized
SEM	Scanning electron microscopy
FT-IR	Fourier-transform infrared spectroscopy
EDL	Electric double-layer
FEA	Finite element analysis
