# Supplementary material for: A Flexible Capacitive Pressure Sensor with Broad-Range High Sensitivity Based on 3D Porous Ionogel for Wearable Health Monitoring
Source: Micromachines (Basel). 2026 Aug 20;17(8):983. doi: 10.3390/mi17080983 (PMC13515964; doi:10.3390/mi17080983)
Supplement: Supplementary file 1 [file micromachines-17-00983-s001.zip › micromachines-4489250-supplementary.pdf]

Supplementary Information:

S1 Qualitative Stress-distribution Analysis

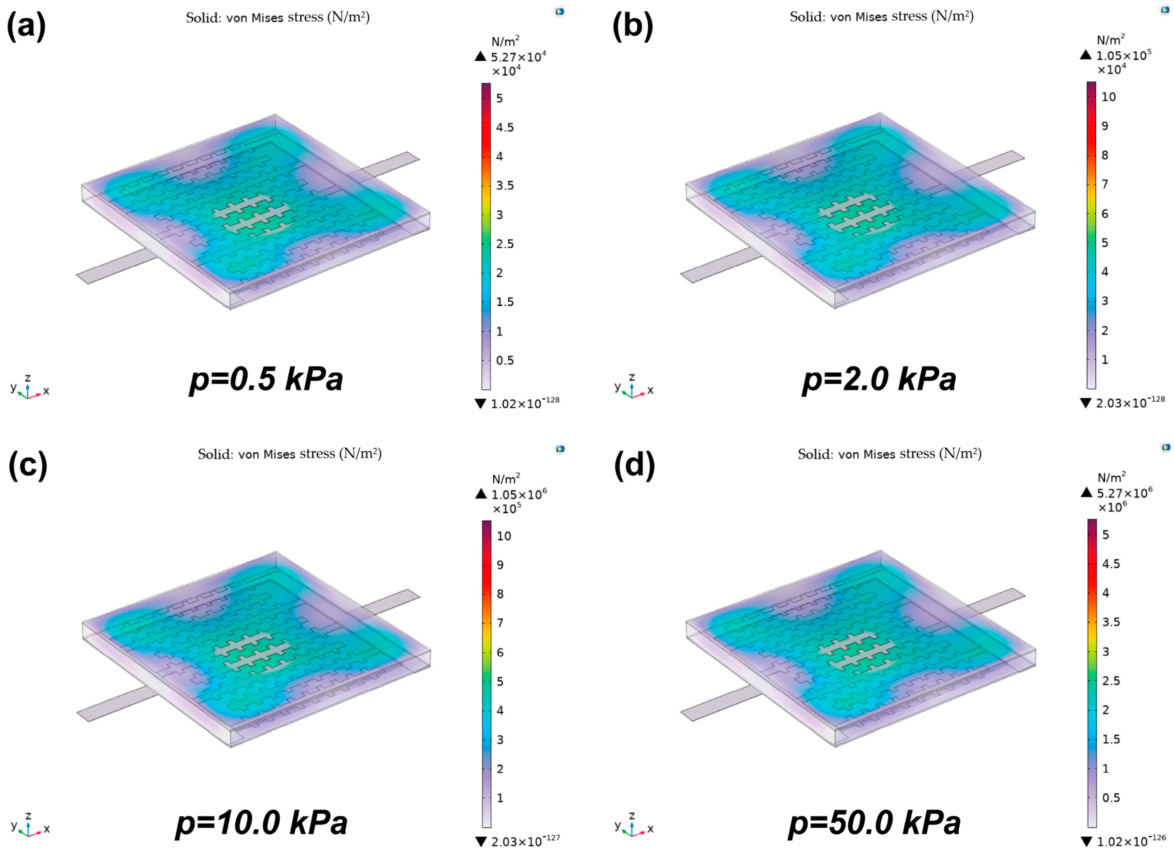

Figure S1. Qualitative stress-distribution analysis of capacitive pressure sensors under different pressures simulation: (a) p=0.5 kPa, (b) p=2.0 kPa, (c) p=10.0 kPa, (d) p=50.0 kPa.

Figure S1 shows the qualitative stress-distribution tendency of the sensor structure under different applied pressures. The simulated stress field shows a non-uniform distribution, with relatively higher stress appearing near the patterned electrode/sensing region. This result is used only to qualitatively visualize the stress-distribution tendency under loading. The actual deformation behavior of the porous ionogel foam is discussed based on the experimentally measured stress-strain curve in Figure 3b of the main text.

Table S1. Absolute capacitance output and normalized capacitance response under different pressures.

Pressure (kPa)	Measured total capacitance, C (nF)	Measured initial capacitance, C_0,total (nF)	Absolute capacitance change, ΔC (nF)	Effective C_0 used for $\Delta C/C_0$ (nF)	$\Delta C/C_0$
0	16.4411	16.4411	0	0.0411	0
0.2	16.7044	16.4411	0.2633	0.0411	7.17
0.5	16.9736	16.4411	0.5325	0.0411	13.62
1	17.9158	16.4411	1.4747	0.0411	36.06
2	19.3228	16.4411	2.8817	0.0411	67.58
5	28.2563	16.4411	11.8152	0.0411	288.36
10	40.1961	16.4411	23.7550	0.0411	572.23
20	51.9160	16.4411	35.4749	0.0411	857.39
50	71.4914	16.4411	55.0503	0.0411	1340.28

Table S2. Capacitance parameters before and after 6000 compression-release cycles.

Parameter	Initial (nF)	After 6000 cycles (nF)	Change or retention
Baseline capacitance	16.4406	16.4406	0 nF
Loaded capacitance	71.4914	71.4914	0 nF
Response amplitude,ΔC	55.0508	55.0508	100.0% retention
